# Associations between neighbourhood street connectivity and sedentary behaviours in Canadian adults: Findings from Alberta’s Tomorrow Project

**DOI:** 10.1371/journal.pone.0269829

**Published:** 2022-06-30

**Authors:** Vikram Nichani, Mohammad Javad Koohsari, Koichiro Oka, Tomoki Nakaya, Ai Shibata, Kaori Ishii, Akitomo Yasunaga, Jennifer E. Vena, Gavin R. McCormack

**Affiliations:** 1 Department of Community Health and Epidemiology, University of Saskatchewan, Saskatoon, Saskatchewan, Canada; 2 Faculty of Sport Sciences, Waseda University, Tokorozawa, Saitama, Japan; 3 Graduate School of Environmental Studies, Tohoku University, Sendai, Miyagi, Japan; 4 Faculty of Health and Sport Sciences, University of Tsukuba, Tsukuba, Ibaraki, Japan; 5 Faculty of Liberal Arts and Sciences, Bunka Gakuen University, Shibuya City, Tokyo, Japan; 6 Department of Community Health Sciences, Cumming School of Medicine, University of Calgary, Calgary, Alberta, Canada; 7 Cancer Care Alberta, Alberta Health Services, Calgary, Alberta, Canada; Public Health Agency of Canada, CANADA

## Abstract

Evidence suggests that neighbourhood street connectivity is positively associated with physical activity, yet few studies have estimated its associations with sedentary behaviour. We estimated the associations between space syntax derived *street integration*, a novel measure of street connectivity, and sedentary behaviours among Canadian adults. Data were sourced from a population-based study–Alberta’s Tomorrow Project (n = 14,758). Items from the International Physical Activity Questionnaire captured sedentary behaviour, including sitting and motor vehicle travel time and walking. *Street integration* was measured within a 1600m radius of participants’ homes. Covariate-adjusted linear regression models estimated the associations between *street integration* and sedentary behaviour. *Street integration* was significantly positively associated with daily minutes of sitting on week (b 6.44; 95CI 3.60, 9.29) and weekend (b 4.39; 95CI 1.81, 6.96) days, and for week and weekend days combined (b 5.86; 95CI 3.30, 8.41) and negatively associated with daily minutes of motor vehicle travel (b -3.72; 95CI -3.86, -1.55). These associations remained significant after further adjustment for daily walking participation and duration. More research is needed to understand the pathways by which street integration positively and or negatively affects sedentary behaviour.

## Introduction

Sedentary behaviour is categorized as any waking activity that causes an energy expenditure of ≤1.5 metabolic equivalents while in sitting or reclining posture [1]. Activities such as watching television, using computers, and travelling in a motor vehicle are considered sedentary behaviours [2]. The prevalence of sedentary behaviour has increased not only in Canada but also in several other countries [3,4]. Current Canadian sedentary behaviour guidelines recommend that people limit their daily sedentary time to less than 8 hours to reduce health risks [5]. Despite this recommendation, Canadian adults, on average, engage in sedentary activities for 9.6 hours/day [6].

Population-level interventions, such as modifying the built environment, may discourage sedentary behaviour [2]. Notably, studies have found that neighbourhood built characteristics such as land use (business destination density), street connectivity (intersection density), population density, and environmental greenness are associated with less sedentary time among adults [7,8]. In particular, street connectivity is important as it provides a foundation or “urban skeleton” that informs decisions about the planning, location, and design of other built characteristics (e.g., residential density, sidewalk and pathway location, and land use allocation and mix) [9]. Higher street connectivity reflects route directness [10], which is characterised by grid-like street patterns, short block sizes, more alternative route options, and fewer dead-ends. In addition to supporting physical activity, a few studies have found higher street connectivity associated with less time spent sitting [11,12] and travelling in motor vehicles [7]. Previously, our team found street connectivity (i.e., the density of 3-way and 4-way intersections within 400m of home) and walkability to be negatively associated with time spent travelling in motor vehicles, however, the density of 3-way intersections (but not 4-way intersections) and walkability were also positively associated with sitting time [13]. Others have also found counter-intuitive findings whereby higher walkability was associated with more sitting [14,15].

To advance our understanding of the relationships between the built environment and physical activity, researchers have begun to explore novel ways of estimating neighbourhood built characteristics [16–19]. Methods used in previous studies to estimate street connectivity, such as intersection density, do not truly reflect the configuration structure of urban form and street layout and may not capture the underlying support for neighbourhood travel [20,21]. Space syntax is a method that focuses on the relational aspect of urban form by considering street layouts’ topology [22]. Space syntax measures of connectivity include street integration, which reflects the number of turns taken by a person to travel from one street to another street within a street network [23]. Several studies have found consistent positive associations between street integration and indicators of pedestrian movement, including leisure and transportation walking [17,20,24]. Few studies in Canada [7] and elsewhere [17,25] however, have explored associations between street integration and sedentary behaviour. For example, Koohsari et al. [7] found that higher space syntax walkability (street integration and population density combined) was associated with lower leisure screen time (weekly hours of watching television or using a computer) among Canadian adults. Studies undertaken in Australia [17] and Japan [25] found that higher street integration was associated with lower odds of driving a car over 60 minutes/day.

Despite evidence showing associations between the neighbourhood built environment and sedentary behaviour, the potential causal mechanisms explaining such associations remain unclear. Explanations of this relationship relate to the potential of the neighbourhood built environment to effect physical activity positively and therefore displace sedentary behaviour or that specific features of the built environment directly influence sedentary behaviour (e.g., lack of transport infrastructure making vehicle travel more convenient) [26,27]. Both explanations appear plausible given the inverse associations between time in physical activity and sedentary behaviour, suggesting one behaviour displaces the other [28]. Notably, other research suggests that adults can be both highly physically active and highly sedentary (i.e., “active couch potato”) [29]. It is plausible that people residing in walkable neighbourhoods who are highly physically active (e.g., walking) might compensate by also undertaking more sedentary behaviour.

The purpose of this study was to estimate the associations between street integration and self-reported sitting and motor vehicle travel times in a sample of Canadian adults. Given that street integration is positively associated with walking [17,20,24], and walking is inversely associated with sitting time [28], we also examined walking as a potential confounder of this relationship.

## Methods and materials

### Data source

We conducted a secondary analysis of sociodemographic and sedentary behaviour data sourced Alberta’s Tomorrow Project (ATP). The ATP objectives and recruitment details are described elsewhere [30,31]. In brief, from 2000 to 2008, adults residing in Alberta (Canada) urban and rural areas (n = 63,486) were asked to complete a health and lifestyle questionnaire (HLQ) [30,31]. Of those contacted, 31,072 Albertans completed the HLQs, thereby providing sociodemographic and health data [30]. In 2008, participants completed a follow-up survey through which new data on sociodemographic, health (including sedentary behaviour), and environment domains were collected [30]. Using these 2008 data, our study sample was comprised of adults residing in urban areas who returned the completed follow-up survey (n = 15,342). The University of Calgary Conjoint Health Research Ethics Board approved this secondary analysis (REB17-1466). ATP participants provided written informed consent, which included the use of their de-identified data for approved research projects.

### Variables

#### Street integration

Consistent with previous studies [7,32], we objectively estimated neighbourhood street integration within a 1600m buffer of participant’s homes. Specifically, we defined “neighbourhood” as a 1600m Euclidian buffered area around each participant’s geocoded 6-digit residential postal code. A 1600m radius represents the approximate distance that can be travelled during a 15–20 minute walk [32]. We used Axwomen (26) and DepthMap (27) software to estimate a street integration score for each buffer. Using 2008 street centreline data, we calculated a street integration score for each street segment after aligning other street segments within a 1600m distance from its centre. Subsequently, the mean street integration score for the buffer was calculated [24].

#### Sedentary behaviour

Two sedentary behaviours were captured using items from the International Physical Activity Questionnaire (IPAQ) [33]. Participants reported their sitting and motor vehicle travel time within the last 7 days [30]. Sitting included time spent sitting at work, home, and during leisure, including watching television on weekdays and weekends but excluded motor vehicle travel [34]. Motor vehicle travel included time spent sitting in a motor vehicle while travelling to work areas, shopping centres, movie theatres and other destinations [34]. We estimated average sitting minutes per day for week days, weekend days, week and weekend days combined, and travel by motor vehicle.

#### Walking behavior

Participants reported the number of days and usual minutes per day walking for transport and leisure in the last 7 days using items from the IPAQ [33]. Using established protocols for calculating IPAQ outputs and reducing over-reporting [35], we estimated and summed the total weekly leisure and transport walking minutes into total weekly walking minutes. We then divided total weekly walking minutes by 7 to estimate the average walking minutes per day to align with the sedentary outcomes. We also categorized average walking minutes per day (any walking: >0 minutes vs. no walking: 0 minutes).

#### Sociodemographic characteristics

We incorporated key sociodemographic covariates [8,36,37] into the analysis. These covariates included age, sex, self-reported general health status, current marital status, number of children in household, highest education level, current employment status, annual household income, and season participants returned the survey.

### Data analysis

Given the low proportion of missing cases (3.8% missing; n = 584), we undertook a complete case analysis. We calculated descriptive statistics for all variables. We first regressed the four sedentary behaviour outcomes on *street integration*, adjusting for sociodemographic covariates using multivariable linear regression. Next, we re-estimated these regression models with walking participation and average walking minutes per day also included as covariates. From the regression models, we estimated unstandardized beta-coefficients (b), robust standard errors, and 95 per cent confidence intervals (95CI). Street integration was standardized (converted to z-scores) prior to the analysis. We considered *p values* less than 0.05 as statistically significant. We undertook the analysis using Stata Version 15 (Stata Corp LLC, Texas, USA).

## Results

### Descriptive statistics for sociodemographic characteristics and sedentary behaviour

Our analysis included 14,758 complete cases. Approximately 62% of the sample was represented by women, 85% were middle-aged, 41% reported very good general health, 77% were married or common-law, 73% had no children under 18 years of age living in the household, 77% had completed some or entire post-secondary education, 54% were full-time workers, and 42% had annual household incomes of >$100,000/year (Table 1). Approximately one-half of respondents completed the questionnaire during autumn. Of the 80.8% of participants who reported any walking, the mean time spent walking was 40.3 (SD 38.5) minutes per day. For walkshed areas, the raw street integration scores ranged from 0 to 560 (mean = 169.3; SD = 86.4; median = 157.0; interquartile range = 107) (Table 1). By comparison, the mean of street integration was 954.6 and 179.7 in two previous studies conducted in Japan [38] and Australia [39], respectively. However, unlike our study that included urban areas only, these two studies included urban and rural areas.

**Table 1:** Descriptive characteristics for participants (n = 14,758).

Variable	mean (SD) or %
**Age (years)** 35 to <45 45 to <55 55 to <65 ≥65	14.7 37.6 30.2 17.5
**Sex** Men Women	38.3 61.7
**Self-reported general health status** Poor or fair Good Very good Excellent	7.6 34.2 41.1 17.1
**Current marital status** Married or common-law Separated or divorced Widowed Single, never married	77.1 12.4 4.6 5.9
**Number of children currently in the household** 0 1 2 ≥3	72.9 12.0 11.1 4.0
**Highest education level** Some or entire high school Some or entire technical college training Some or entire university degree Some or entire university post-graduate degree	22.9 38.3 26.0 12.8
**Current employment status** Working full-time Working part-time Homemaker Retired Other or not employed or student	53.6 14.5 6.4 20.7 4.8
**Annual household income** $0 to 49,999 $50,000 to 99,999 $100,000 to 149,999 $150,000 to 199,999 $200,000 to 249,999 $≥250,000 Refused to answer	18.9 31.7 22.9 9.5 4.0 5.2 7.8
**Season of receipt of the survey** Winter Spring Summer Autumn	19.5 4.5 25.2 50.8
**Street integration** *	169.3 (86.4)
**Walking participation (any)**	80.8
**Walking duration (mean min/day)** **	40.3 (38.5)

**Abbreviation**: SD = standard deviation.

*Street integration estimated in a 1600m buffer around each participant’s household address.

**Excludes participants reporting no walking participation.

### Associations between street integration and sitting time

On average, participants reported sitting 336.9 (SD = 181.5) minutes/day on week days and 291.6 (SD = 160.0) minutes/day on weekend days (Table 2). Adjusting for sociodemographic covariates, higher street integration was associated (p < .01) with more time sitting on the average week day (b 6.44; 95CI 3.60, 9.29), weekend day (b 4.39; 95CI 1.81, 6.96), and for week and weekend days combined (b 5.86; 95CI 3.30, 8.41) (Table 3). Notably, the association between street integration and these three sitting outcomes strengthened slightly and remained significant (p < .001) after further adjustment for walking participation and walking duration. Higher walking was significantly negatively associated with the three sitting outcomes (p < .01) (Table 3).

**Table 2:** Sedentary behaviours for participants of Alberta’s Tomorrow Project (n = 14,758).

Types of behaviours	Mean (Standard Deviation) / median
Time spent sitting on weekdays (minutes/day)	336.9 (181.5) / 300
Time spent sitting on weekend days (minutes/day) (minutes/day)	291.6 (160.0) / 240
Time spent sitting on weekdays and weekends (minutes/day) ((minutes(minutes/day)	330.0 (160.7) / 300
Time spent travelling in a motor vehicle (minutes/day)	69.3 (68.2) / 60

**Table 3:** Associations between street integration, sedentary behaviour, and walking for participants of Alberta’s Tomorrow Project.

	Time spent sitting on weekdays (minutes/day)	Time spent sitting on weekends (minutes/day)	Time spent sitting on weekdays and weekends (minutes/day)	Time spent travelling in a motor vehicle (minutes/day)
	b (95% CI)	b (95% CI)	b (95% CI)	b (95% CI)
Street integration^a^	6.44 (3.60, 9.29)**	4.39 (1.81, 6.96)*	5.86 (3.30, 8.41)**	-2.71 (-3.86, -1.55)**
Street integration^b^	6.67 (3.82, 9.52)**	4.94 (2.37, 7.51)**	6.18 (3.62, 8.73)**	-2.66 (-3.81, -1.51)**
Walking (any vs. none)^b^	-12.38 (-20.10, -4.47)*	-29.40 (-36.41, -22.40)**	-17.18 (-24.17, -10.18)**	-2.37 (-5.50, 0.77)
Street integration^c^	6.67 (3.62 9.72)**	4.70 (1.95, 7.45)*	6.11 (3.38, 8.83)**	-2.79 (-4.00, -1.57)**
Walking (minutes/day)^c^	-0.56 (-0.63, -0.48)**	-0.42 (-0.48, -0.35)**	-0.52 (-.0.58, -0.45)**	0.13 (0.10, 0.16)**

a = adjusted for age, sex, self-reported general health, current marital status, number of children in household, highest education level, current employment status, annual household income, and season of the receipt of the survey (n = 14,758).

b = adjusted for age, sex, self-reported general health, current marital status, number of children in household, highest education level, current employment status, annual household income, the season of the receipt of the survey, and walking duration (n = 14,758).

c = adjusted for age, sex, self-reported general health, current marital status, number of children in household, highest education level, current employment status, annual household income, the season of the receipt of the survey, and walking duration (among walkers only; n = 11,924).

Street integration values are standardized (z scores).

Neighbourhood defined as a 1600m buffered area around each participant’s household address.

* = p-value < .01.

** = p-value < .001.

### Associations between street integration and motor vehicle travel time

On average, participants reported travelling in a motor vehicle for 69.3 (SD = 68.2) minutes/day (Table 2). Adjusting for sociodemographic covariates, higher street integration was associated (p < .001) with less time per day spent travelling by motor vehicle (b -2.71; 95CI -3.86, -1.55) (Table 3). The association between street integration and daily motor vehicle travel time remained relatively unchanged and significant (p < .001) after adding walking participation and walking duration as covariates (Table 3). Notably, walking minutes, but not walking participation, was significantly (p < .001) positively associated with daily motor vehicle travel time (Table 3).

## Discussion

Our study examined the associations between a space syntax measure of connectivity (i.e., street integration) and different types of sedentary behaviour. In support of the notion that different sedentary behaviours have specific determinants [40], we found that a one standard deviation increase in street integration, estimated within 1600m of participant’s homes, was on average, associated with a 6.67 minute/day increase in sitting on week days, a 4.70 minute/day increase in sitting on weekend days, a 6.11 minute/day increase in sitting on week and weekend days combined, and a 2.79 minute/day decrease in motor vehicle travel time. A unique finding was that the associations between street integration and the sedentary behaviour outcomes did not substantially change and remained significant after adjusting for walking behaviour. Despite the modest associations found between street integration and sedentary behaviour, these associations are important from urban design and public health perspective given that time spent in motor vehicles [41,42] and time spent sitting [43,44] are risk factors for poorer health outcomes.

Higher space syntax walkability has been reported to be associated with less leisure-based screen time [7]. However, this previous study included a smaller Canadian sample from one city and did not examine street integration alone but instead combined street integration with population density into an index. Our measure of sitting captured overall sitting across different contexts (i.e., work, home, and during leisure) yet neighbourhood street integration still emerged as a significant correlate. Walkability indices that have included indicators of street connectivity have also found positive associations with sitting time [13–15], although these studies did not statistically control for physical activity. Our findings suggest the positive associations between street integration and sitting time is independent of walking and therefore may result from a mechanism that does not involve walking. Speculatively, neighbourhoods with higher street connectivity often have a higher density of local destinations [45], some of which may encourage sitting (e.g., cafes and restaurants). Thus the positive associations between street integrations and sitting may be the result of the neighbourhood also having higher availability to destinations. While this explanation aligns with other evidence demonstrating positive associations between street integration and walking [17,20,24], it is contradicted by a previous study with our sample that found a negative association between the count of business destinations within a 400m radius of home and sitting time [28]. The mechanisms by which street integration is associated with sitting cannot be determined from our study; nevertheless, our findings suggest (re)designing neighbourhoods with higher street integration may be associated with increased time spent sitting on week and weekend days. Neighbourhood socioeconomic status, occupation-related physical activity or working hours, and additional built environment variables, among other potential correlates [8], may need to be examined as potential confounders in future studies to better understand the associations between street integration and sitting.

Consistent with previous studies [17,25], we found that higher street integration was associated with less time spent on motor vehicle travel. For example, among a sample of Australian adults, Koohsari et al. [17] found that street integration was associated with lower odds of driving a car more than 60 minutes/day. A Japanese study also reported that higher street integration was associated with lower odds of driving a car more than 60 minutes/day [25]. Others have found neighbourhood street connectivity, measured using non-space syntax approaches, to be negatively associated with driving time [7]. Street integration is a measure of network connectivity, which reflects the feasibility of travelling from one destination to another or the ability to travel through the neighbourhood conveniently. Street integration [17,19] and other measures of street connectivity (43) are associated with transportation walking. Thus, in neighbourhoods with high street connectivity, compared with driving, transportation walking might be a more convenient method of local travel. However, we found that the negative association between street integration and time spent travelling in a motor vehicle remained relatively unchanged and significant even after statistically controlling for walking (which included transport and leisure walking). More connected neighbourhoods typically have more utilitarian destinations (e.g., cafes, shops) [21], which may encourage transportation walking [46,47] however, it may also result in shorter motor vehicle trips and thus less time spent in motor vehicles among those who continue to drive to these potentially walkable destinations [48]. Building neighbourhoods with higher street integration could provide other population health benefits, as less time spent in motor vehicles might reduce air pollution [49,50] and the incidence of vehicle-related pedestrian and cyclist injuries [51].

Our study’s strengths include the large, diverse sample of sociodemographic characteristics and urban geographical locations, the estimation of a novel objective measure of neighbourhood connectivity (street integration), and the inclusion of two different sedentary behaviours (sitting time and motor vehicle travel time). However, our study has some limitations. We cannot infer causality from these cross-sectional data. Our analysis adjusted for sociodemographic characteristics and walking; however, our estimates did not account for people’s reasons for moving to their neighbourhood (e.g., behavioural tendencies that inform residential choices) or control for other neighbourhood built or socioeconomic characteristics. Other built environment variables measured within the 1600m buffer were not available. Self-reported sedentary time may not accurately reflect actual sedentary time [52]. Moreover, our measure of overall sitting was neither context nor domain-specific, thus, making it difficult to isolate sitting time undertaken in the neighbourhood (e.g., at home or local destinations). This lack of specificity may have resulted in estimated associations between street integration and sitting that were smaller in magnitude. Despite these limitations, our findings provide useful evidence to inform future studies that might use more rigorous methods (e.g., longitudinal studies or natural experiments that incorporate context-specific accelerometer-measured sedentary behaviour) to investigate the relations between the built environment and sedentary behaviour.

Our findings suggest that street integration is associated with different sedentary behaviours, albeit in different directions. While the mechanism(s) by which street integration is associated with sitting time remains unexplained, these associations are independent of walking behaviour and warrant further investigation. Urban design and public health professionals and researchers need to consider the different pathways by which neighbourhood built characteristics positively and negatively affect sedentary and other health behaviours.
